# Severe Lithium Toxicity Following Concurrent Lithium and Nabumetone Use in a Patient With Bipolar I Disorder: A Case Report

**DOI:** 10.7759/cureus.112142

**Published:** 2026-07-06

**Authors:** Michael R Hower, Holly Agud, Prasad R Padala

**Affiliations:** 1 Psychiatry Residency Program, Baptist Health – University of Arkansas for Medical Sciences (UAMS), North Little Rock, USA; 2 Geriatric Research Education and Clinical Center, Central Arkansas Veterans Healthcare System, Little Rock, USA

**Keywords:** adverse drug reaction, bipolar disorder, consultation-liaison psychiatry, lithium toxicity, nabumetone, naranjo scale, nsaid interaction, renal lithium clearance

## Abstract

Lithium remains a cornerstone in the treatment of bipolar I disorder because of its well-established efficacy in reducing mood episode recurrence and suicide risk. However, its narrow therapeutic index makes patients particularly susceptible to clinically significant toxicity, especially following medication changes or drug-drug interactions. Nonsteroidal anti-inflammatory drugs (NSAIDs) are recognized to increase serum lithium concentrations by reducing renal lithium clearance, although reports involving nabumetone remain limited. We report the case of a 50-year-old woman with bipolar I disorder who had remained psychiatrically stable on chronic lithium therapy for approximately seven years before developing severe lithium toxicity following recent escalation of both lithium and nabumetone dosages. The patient presented with progressive confusion, lethargy, recurrent falls, tremor, myoclonic jerks, gait instability, generalized weakness, and altered mental status. Initial serum lithium concentration exceeded 2.9 mmol/L despite preserved renal function. Lithium and nabumetone were discontinued, aggressive intravenous fluid administration was initiated, and serial lithium concentrations were monitored. Given preserved renal function, declining serum lithium concentrations, and rapid neurologic improvement with supportive management, hemodialysis was deferred. The patient experienced progressive clinical recovery with normalization of lithium concentrations and resolution of neurologic symptoms. Lithium was cautiously reintroduced at a reduced dosage without recurrence of toxicity. Application of the Naranjo Adverse Drug Reaction Probability Scale yielded a score of 9, supporting a definite adverse drug reaction. This case highlights that severe lithium toxicity may occur despite preserved renal function following concurrent escalation of lithium and nabumetone therapy. The report emphasizes the importance of comprehensive medication reconciliation, recognition of clinically significant drug-drug interactions, close serum lithium monitoring following medication changes, and early recognition of neurologic manifestations suggestive of lithium toxicity.

## Introduction

Bipolar I disorder is a chronic psychiatric illness characterized by recurrent episodes of mania and depression that frequently require long-term pharmacologic treatment to prevent relapse and reduce morbidity [1]. Lithium remains one of the most effective maintenance therapies for bipolar I disorder and has consistently demonstrated efficacy in reducing mood episode recurrence while also lowering suicide risk [2,3]. Despite its long history of clinical use, lithium presents unique management challenges because of its narrow therapeutic index and susceptibility to clinically significant drug-drug interactions [2,4,5].

The therapeutic serum lithium concentration for maintenance therapy generally ranges from approximately 0.6 to 1.2 mmol/L. Concentrations exceeding this range substantially increase the risk of toxicity, although clinical manifestations correlate imperfectly with serum levels [2,5]. Lithium is almost exclusively eliminated through renal excretion, making serum concentrations highly dependent upon renal function, sodium balance, hydration status, and concomitant medications that alter renal lithium clearance [2,4,5]. Consequently, relatively modest physiologic changes or medication adjustments may result in clinically significant increases in serum lithium concentrations.

Lithium toxicity commonly presents with neurologic manifestations including confusion, lethargy, tremor, ataxia, dysarthria, myoclonus, gait instability, hyperreflexia, altered mental status, and, in severe cases, seizures or coma [2,5]. Because these symptoms are often nonspecific, prompt recognition requires careful clinical assessment, thorough medication reconciliation, and measurement of serum lithium concentrations.

Numerous medications have been associated with elevated lithium concentrations, including angiotensin-converting enzyme inhibitors, angiotensin receptor blockers, thiazide diuretics, and nonsteroidal anti-inflammatory drugs (NSAIDs) [3]. NSAIDs decrease renal prostaglandin synthesis, reducing renal perfusion and lithium clearance [4,6,7]. Although this interaction is well recognized, published reports specifically describing severe lithium toxicity associated with nabumetone remain limited [8,9]. Nabumetone possesses relative cyclooxygenase-2 selectivity compared with traditional NSAIDs but still has the potential to impair renal lithium elimination sufficiently to produce clinically significant toxicity [4,6-9].

We present the case of a patient with bipolar I disorder who remained psychiatrically stable on lithium therapy for approximately seven years before developing severe lithium toxicity following concurrent escalation of both lithium and nabumetone therapy. Despite preserved renal function throughout hospitalization, she developed marked neurologic manifestations requiring inpatient management. This case highlights the importance of recognizing clinically significant lithium-NSAID interactions, performing meticulous medication reconciliation following medication changes, and maintaining close serum lithium monitoring whenever medications capable of altering lithium pharmacokinetics are initiated or adjusted [6,7,10,11-13].

## Case presentation

A 50-year-old woman with bipolar I disorder presented to the emergency department with progressive confusion, lethargy, recurrent falls, tremor, gait instability, and declining responsiveness following recent outpatient adjustments to both lithium and chronic NSAID therapy. She had remained psychiatrically stable on maintenance lithium therapy for approximately seven years prior to presentation without any documented episodes of clinically significant lithium toxicity.

Her past medical history was notable for bipolar I disorder, hypothyroidism treated with levothyroxine 50 mcg daily, chronic low back pain managed with nabumetone, gastroesophageal reflux disease, hyperlipidemia, and type 2 diabetes mellitus. She had no documented history of chronic kidney disease, and previous laboratory monitoring demonstrated preserved renal function. Home medications included atorvastatin 40 mg daily, baclofen 10 mg three times daily, bupropion XL 150 mg daily, gabapentin 300 mg three times daily, levothyroxine 50 mcg daily, metformin XR 500 mg daily, nortriptyline 20 mg nightly, omeprazole 20 mg daily, propranolol 20 mg twice daily, venlafaxine XR 75 mg daily, ziprasidone 80 mg twice daily, lithium, and nabumetone.

Approximately one month before admission, lithium dosage had been increased from 900 mg/day (300 mg each morning and 600 mg nightly) to 1,200 mg/day (600 mg twice daily) to improve mood stabilization. Several weeks before hospitalization, nabumetone dosage was also increased from 1,500 mg/day (750 mg twice daily) to 2,000 mg/day (500 mg four times daily). Celecoxib 200 mg twice daily had also been prescribed but had reportedly not been taken for approximately one month before presentation. Tizanidine had been prescribed on an as-needed basis but had likewise not been taken during the week preceding admission.

Although one historical outpatient lithium concentration measured approximately seven years before presentation was reported as 1.5 mmol/L, this isolated value was not associated with symptoms of toxicity, hospitalization, medication adjustment, or documented adverse effects. Subsequent outpatient lithium concentrations remained within or near the accepted maintenance therapeutic range (approximately 0.6-0.7 mmol/L), and the patient remained psychiatrically stable on chronic lithium therapy for several years without evidence of clinically significant toxicity. 

Over several weeks preceding hospitalization, family members noted recurrent falls, worsening balance, increasing fatigue, and progressive cognitive slowing. During the week before admission, the patient developed worsening confusion, decreased responsiveness, generalized weakness, progressive tremor, and increasing difficulty ambulating. On the day before the presentation, she became minimally verbal, prompting emergency medical evaluation.

Initial examination demonstrated an ill-appearing woman with altered mental status. She was somnolent but arousable, oriented only intermittently, and followed only simple commands. Speech was slowed and limited by decreased responsiveness. Cranial nerves were grossly intact without focal deficits. Motor examination demonstrated generalized weakness without lateralizing findings. A coarse postural tremor was present bilaterally, together with multifocal myoclonic jerks involving the upper extremities. Marked gait instability and truncal ataxia prevented safe independent ambulation. Deep tendon reflexes were mildly increased diffusely without sustained clonus. No meningeal signs or focal neurologic deficits suggestive of an acute cerebrovascular event were identified.

The differential diagnosis included acute lithium toxicity, medication-induced encephalopathy related to polypharmacy, serotonin syndrome, toxic-metabolic encephalopathy, acute cerebrovascular disease, and seizure disorder. However, the combination of recent lithium dose escalation, concurrent increase in nabumetone dosage, characteristic neurologic findings, markedly elevated serum lithium concentration, preserved renal function, and rapid clinical improvement following discontinuation of lithium and aggressive intravenous hydration strongly supported lithium toxicity as the primary diagnosis. Serotonin syndrome was considered less likely because the patient lacked sustained hyperthermia, marked autonomic instability, inducible or spontaneous clonus, and severe neuromuscular hyperactivity characteristic of that syndrome [2,5]. Acute stroke was considered unlikely given the absence of focal neurologic deficits and subsequent clinical improvement without stroke-directed intervention.

Initial laboratory evaluation demonstrated a serum lithium concentration greater than 2.9 mmol/L. Serum creatinine was 0.95 mg/dL (reference range 0.57-1.11 mg/dL), estimated glomerular filtration rate was 73 mL/minute/1.73 m², and calculated creatinine clearance was approximately 120 mL/min, indicating preserved renal function. Urine drug screening was positive for cannabinoids. No clinically significant electrolyte abnormalities were identified that adequately explained the patient's presentation. Additional laboratory findings are summarized in Table 1.

**Table 1:** Clinical Timeline and Key Laboratory Findings QAM: *quaque ante meridiem* (every morning); QHS: *quaque hora somni* (every night at bedtime); BID: *bis in die* (twice a day)

Time Point	Clinical Findings	Serum Lithium (mmol/L)	Renal Function / Laboratory Findings	Medication Changes / Management
~7 years prior to admission	Psychiatrically stable on maintenance lithium therapy. An isolated serum lithium concentration of 1.5 mmol/L was documented without symptoms of toxicity, hospitalization, medication adjustment, or other clinical intervention.	1.5	No evidence of renal dysfunction documented.	Continued lithium 600 mg twice daily.
~6 years prior to admission	Continued psychiatric stability on maintenance lithium therapy.	0.6–0.7	Stable renal function.	Continued lithium 600 mg twice daily.
Approximately 1 month prior to admission	No symptoms of lithium toxicity.	—	—	Lithium increased from 900 mg/day (300 mg QAM, 600 mg QHS) to 1200 mg/day (600 mg BID).
Several weeks prior to admission	No symptoms of lithium toxicity immediately following medication adjustment.	—	—	Nabumetone increased from 1500 mg/day (750 mg BID) to 2000 mg/day (500 mg QID).
Hospital Day 1 – Presentation	Somnolence, confusion, lethargy, tremor, multifocal myoclonic jerks, generalized weakness, gait instability, inability to ambulate independently, recurrent falls, altered mental status.	2.93	Creatinine 0.95 mg/dL (0.57–1.11), eGFR 73 mL/min/1.73 m², creatinine clearance approximately 120 mL/min, urine drug screen positive for cannabinoids.	Lithium and nabumetone discontinued. Aggressive intravenous isotonic fluids initiated. Serial lithium concentrations and neurologic examinations performed.
Following initial IV fluid administration	Rapid improvement in alertness and mentation. Persistent but improving tremor and myoclonic movements.	2.63	Renal function remained preserved.	Continued supportive management. Hemodialysis deferred because of preserved renal function, declining lithium concentrations, and improving neurologic examination.
During hospitalization	Progressive improvement in mental status, gait, tremor, and myoclonic jerks.	1.70	Stable renal function throughout hospitalization.	Continued intravenous hydration and serial laboratory monitoring.
Hospital Day 3	Clinically improved with marked neurologic recovery.	1.12	Stable renal function.	Lithium cautiously restarted at 300 mg twice daily.
Hospital Day 5 – Discharge	Returned to baseline mental status with resolution of confusion and myoclonic jerks and marked improvement in gait stability.	0.82	Stable renal function.	Nabumetone remained discontinued. Outpatient lithium monitoring and medication management recommended.

Neither the patient nor the family reported significant vomiting, diarrhea, febrile illness, or other acute medical conditions immediately preceding hospitalization. Although decreased oral intake likely occurred as neurologic symptoms progressed, no alternative precipitating factor sufficiently accounted for the abrupt onset of severe toxicity beyond the recent concurrent escalation of lithium and nabumetone therapy.

Lithium and nabumetone were discontinued immediately upon diagnosis. Aggressive intravenous isotonic fluid administration was initiated together with serial neurologic examinations and frequent serum lithium measurements. Current recommendations regarding extracorporeal treatment for lithium poisoning were considered throughout hospitalization [12,13]. However, because renal function remained preserved, serum lithium concentrations declined appropriately with supportive management, and the patient's neurologic examination demonstrated rapid clinical improvement over the first several hours of treatment, hemodialysis was not pursued [12,13].

Within hours of intravenous fluid administration, mental status improved substantially, progressing from obtundation to conversational responsiveness. Tremor and myoclonic movements gradually diminished over the subsequent hospital days, while gait instability and generalized weakness improved in parallel with declining serum lithium concentrations. Renal function remained stable throughout hospitalization without evidence of acute kidney injury.

Following normalization of serum lithium concentration to 0.82 mmol/L and significant neurologic recovery, lithium therapy was cautiously restarted at a reduced dosage of 300 mg twice daily. At discharge, the patient's mental status had returned to baseline with complete resolution of confusion and myoclonic jerks, marked improvement in gait stability, and no residual focal neurologic deficits. Nabumetone remained discontinued, and the patient was instructed to follow up closely with her outpatient psychiatric provider for serial lithium monitoring and ongoing medication management. The risks of recurrent lithium toxicity associated with NSAID therapy were reviewed extensively, and alternative pain management strategies were recommended [11-13].

Application of the Naranjo Adverse Drug Reaction Probability Scale yielded a score of 9, supporting a definite adverse drug reaction and strengthening the likelihood that the interaction between lithium dose escalation and concurrent nabumetone dosage increase was the principal contributor to the observed toxicity [2].

## Discussion

This case describes severe lithium toxicity following recent concurrent escalation of lithium and nabumetone therapy in a patient with bipolar I disorder who had remained psychiatrically stable on chronic lithium treatment for approximately seven years. Following dose escalation of both medications, the patient developed progressive confusion, lethargy, recurrent falls, tremor, myoclonic jerks, gait instability, generalized weakness, and altered mental status accompanied by a serum lithium concentration exceeding 2.9 mmol/L. Although lithium toxicity has been extensively described in association with NSAID therapy, published reports specifically implicating nabumetone remain limited [8,9]. This case therefore contributes additional evidence supporting the potential for clinically significant lithium accumulation following concomitant lithium and nabumetone dose escalation while emphasizing that toxicity may occur even in the setting of preserved renal function.

Lithium possesses a narrow therapeutic index, and numerous physiologic and pharmacologic factors may increase serum lithium concentrations [2,4,5]. Well-recognized precipitants include dehydration, sodium depletion, acute kidney injury, advanced age, and medications that impair renal lithium elimination [2,5]. Among these medications, NSAIDs, angiotensin-converting enzyme inhibitors, angiotensin receptor blockers, and thiazide diuretics have consistently been associated with increased serum lithium concentrations through reductions in renal lithium clearance [3,11,13]. Consequently, careful medication reconciliation remains essential whenever patients receiving chronic lithium therapy undergo medication changes [11-13].

The biologic mechanism underlying the interaction between lithium and NSAIDs is well established [4,6,7]. NSAIDs inhibit cyclooxygenase-mediated prostaglandin synthesis, resulting in reduced renal prostaglandin production and diminished afferent arteriolar vasodilation. The resulting reduction in renal perfusion decreases lithium clearance, thereby increasing serum lithium concentrations [4,6,7]. Nabumetone is a prodrug that is converted to the active metabolite 6-methoxy-2-naphthylacetic acid (6-MNA) and demonstrates relative cyclooxygenase-2 selectivity compared with traditional NSAIDs [4,8]. Although relative COX-2 selectivity has historically been associated with a lower incidence of gastrointestinal adverse effects, it does not eliminate clinically important renal prostaglandin inhibition [6,7]. Consequently, nabumetone retains the potential to reduce renal lithium clearance sufficiently to precipitate clinically significant toxicity in susceptible individuals [6-9].

Previous reports have demonstrated substantial increases in serum lithium concentrations with other relatively selective COX-2 inhibitors, including celecoxib and rofecoxib [7-9]. Post-marketing surveillance and published case reports have documented marked lithium accumulation accompanied by neurologic manifestations such as tremor, ataxia, confusion, dysarthria, lethargy, and gait disturbance [7-9]. Although comparable reports involving nabumetone remain relatively uncommon, the pharmacologic mechanism is biologically plausible and consistent with the clinical course observed in this patient [8,9]. The close temporal relationship between concurrent dose escalation of lithium and nabumetone, development of characteristic neurologic findings, markedly elevated serum lithium concentration, and subsequent clinical improvement following withdrawal of both medications strongly supports a clinically significant drug-drug interaction.

One of the most clinically important aspects of this case is that severe lithium toxicity occurred despite preserved renal function throughout hospitalization. Lithium toxicity is frequently associated with acute kidney injury or chronic renal impairment; however, preserved serum creatinine, estimated glomerular filtration rate, and calculated creatinine clearance do not exclude clinically significant reductions in lithium clearance [10]. NSAID-induced alterations in renal hemodynamics may impair lithium elimination before measurable abnormalities in conventional markers of renal function become apparent [6,7,10]. This observation highlights an important clinical teaching point: clinicians should avoid relying solely on serum creatinine or estimated glomerular filtration rate when assessing lithium toxicity risk after initiation or dose escalation of interacting medications. Instead, changes in medication regimen, neurologic examination findings, and serial serum lithium concentrations should collectively guide clinical decision-making [10,11].

Another notable feature of this case is the patient's prolonged history of stable lithium therapy before the development of toxicity. Although one isolated historical serum lithium concentration of 1.5 mmol/L had been documented years earlier, it was not associated with clinical manifestations of toxicity, hospitalization, or treatment modification, and subsequent lithium concentrations remained within or near the accepted maintenance therapeutic range while the patient remained psychiatrically stable. This pattern suggests that long-term tolerance of lithium therapy does not eliminate the possibility of future toxicity when interacting medications are introduced or their dosages are increased. Rather than representing chronic lithium intolerance, the patient's clinical course supports an acute pharmacokinetic interaction temporally associated with recent escalation of both lithium and nabumetone therapy.

Current recommendations regarding extracorporeal treatment for lithium poisoning were considered throughout hospitalization [12,13]. Although hemodialysis represents the treatment of choice for selected patients with severe lithium toxicity, the decision to defer dialysis in this case was supported by several favorable clinical factors. The patient maintained preserved renal function throughout hospitalization, serum lithium concentrations declined appropriately following aggressive intravenous hydration, and neurologic status improved rapidly with supportive care alone. Taken together, these findings suggested that conservative management remained appropriate without exposing the patient to the additional risks associated with extracorporeal therapy [12,13]. This case illustrates the importance of individualized clinical assessment in conjunction with established treatment recommendations rather than reliance on serum lithium concentration alone when determining the need for hemodialysis.

Several additional factors were considered during the evaluation of this patient's presentation. Although concomitant medications including baclofen, gabapentin, nortriptyline, venlafaxine, and cannabinoids may contribute to sedation, psychomotor slowing, or altered mental status, these agents would not be expected to produce the characteristic combination of markedly elevated serum lithium concentration, coarse tremor, multifocal myoclonic jerks, progressive gait instability, and subsequent rapid clinical improvement following discontinuation of lithium and aggressive intravenous hydration. Likewise, serotonin syndrome was considered less likely because the patient lacked the constellation of hyperthermia, marked autonomic instability, and sustained inducible or spontaneous clonus typically associated with that diagnosis [2,5]. Acute cerebrovascular disease was also considered but was not supported by the absence of focal neurologic deficits and the patient's rapid clinical improvement with supportive management. Taken together, the temporal association between medication changes, objective laboratory findings, characteristic neurologic manifestations, and clinical response strongly supported lithium toxicity as the primary diagnosis.

Although dehydration represents a well-recognized precipitating factor for lithium toxicity [2,5], neither the patient nor the family reported significant vomiting, diarrhea, febrile illness, or any other acute medical condition immediately preceding hospitalization. Some reduction in oral intake likely occurred as neurologic symptoms progressed; however, this was more likely a consequence of evolving toxicity than its primary cause. While it is impossible to completely exclude a contributory role for reduced oral intake, the close temporal relationship between concurrent lithium and nabumetone dose escalation provides the most plausible explanation for the observed toxicity. Clinicians should nevertheless recognize that lithium toxicity is often multifactorial, with interacting medications, hydration status, sodium balance, and individual patient characteristics collectively influencing lithium clearance [2,5,11].

Application of the Naranjo Adverse Drug Reaction Probability Scale yielded a score of 9, indicating a definite adverse drug reaction. Several components contributed to this assessment, including the temporal relationship between medication escalation and symptom onset, objective evidence of toxicity through markedly elevated serum lithium concentrations, clinical improvement following withdrawal of the suspected medications, and the absence of a more convincing alternative diagnosis. Although the Naranjo scale cannot establish causality independently, it provides a standardized framework that strengthens the overall assessment when interpreted alongside the patient's clinical course and pharmacologic plausibility [2].

This case has several important clinical implications. First, medication reconciliation should extend beyond identifying current medications to include careful review of recent medication additions and dosage adjustments, particularly in patients receiving lithium [11-13]. Second, clinicians should recognize that severe lithium toxicity may occur despite apparently preserved renal function, emphasizing that normal serum creatinine values do not eliminate the need for clinical vigilance [10,11]. Third, patients prescribed chronic lithium therapy should receive counseling regarding medications known to increase lithium concentrations, including NSAIDs, angiotensin-converting enzyme (ACE) inhibitors, angiotensin receptor blockers, and thiazide diuretics [3,11-13]. Finally, serum lithium concentrations should be reassessed promptly following initiation, discontinuation, or dose escalation of medications capable of altering lithium pharmacokinetics, even in patients who have previously demonstrated long-term stability on lithium therapy [11-13].

This report also highlights the importance of communication among healthcare providers when multiple clinicians participate in medication management. In this case, adjustments to lithium and nabumetone therapy occurred within a relatively short interval, illustrating how independently appropriate prescribing decisions may collectively increase the risk of clinically significant drug-drug interactions. Comprehensive medication reconciliation, coordinated communication between treating clinicians, and patient education regarding potentially interacting medications remain critical strategies for reducing preventable adverse drug events [11-13].

Several limitations should be acknowledged. As with any single case report, definitive causality between nabumetone dose escalation and lithium toxicity cannot be established. Although the temporal association, biologic plausibility, objective laboratory findings, Naranjo score, and clinical improvement following discontinuation strongly support a clinically significant interaction, additional factors-including reduced oral intake and concomitant centrally acting medications-may have contributed to the patient's presentation. Furthermore, because reports specifically describing nabumetone-associated lithium toxicity remain limited [8,9], broader conclusions regarding the magnitude of risk should be interpreted cautiously until additional cases become available. Nevertheless, this report contributes meaningful clinical evidence supporting careful monitoring when lithium and nabumetone are prescribed concurrently, particularly following dosage adjustments.

Overall, this case reinforces several important clinical principles. Clinicians should maintain a high index of suspicion for lithium toxicity whenever new neurologic symptoms develop after medication changes, regardless of apparently preserved renal function. Careful medication reconciliation, early recognition of characteristic neurologic manifestations, prompt measurement of serum lithium concentrations, and timely supportive management remain essential for optimizing patient outcomes. As additional reports emerge, a more comprehensive understanding of the interaction between lithium and nabumetone may further refine monitoring recommendations and improve patient safety.

## Conclusions

This case describes severe lithium toxicity following recent concurrent escalation of lithium and nabumetone therapy in a patient with bipolar I disorder who had previously remained psychiatrically stable on chronic lithium treatment for several years. It demonstrates that clinically significant lithium toxicity may occur despite preserved renal function when interacting medications are introduced or their dosages are increased. The temporal association between concurrent medication escalation, characteristic neurologic manifestations, markedly elevated serum lithium concentrations, favorable dechallenge following discontinuation of lithium and nabumetone, and a Naranjo Adverse Drug Reaction Probability Scale score of 9 strongly supports a clinically significant drug-drug interaction. Nevertheless, as with any single case report, definitive causality cannot be established, and additional clinical reports are needed to better characterize the magnitude of risk associated with concomitant nabumetone therapy.

This case reinforces several important clinical principles. Medication reconciliation should include a careful review of recent medication additions and dosage adjustments; clinicians should recognize that normal renal function does not exclude the possibility of severe lithium toxicity, and serum lithium concentrations should be reassessed whenever potentially interacting medications are initiated, discontinued, or adjusted. Early recognition of neurologic manifestations, prompt discontinuation of offending medications, aggressive supportive management, and close laboratory monitoring remain essential to optimizing clinical outcomes. Increased awareness of potentially underrecognized interactions between lithium and relatively selective NSAIDs such as nabumetone may help reduce preventable adverse drug events and improve the safe long-term management of patients receiving chronic lithium therapy.
